# A Rare Case of Bronchial Anthracofibrosis with Pleural Anthracosis Presented as Recurrent Pleural Effusion

**DOI:** 10.1155/2019/2982763

**Published:** 2019-08-28

**Authors:** Rajesh Kumar Yadav, Jitendra Kumar Saini, Prabhpreet Sethi, Rohit Sarin

**Affiliations:** ^1^Senior Resident, Department of TB and Respiratory Diseases, National Institute of TB and Respiratory Diseases, New Delhi, India; ^2^Chest Specialist Grade-I, Department of TB and Respiratory Diseases, National Institute of TB and Respiratory Diseases, New Delhi, India; ^3^Director & Professor, Department of TB and Respiratory Diseases, National Institute of TB and Respiratory Diseases, New Delhi, India

## Abstract

A fifty-eight-year-old, nonsmoker Indian male presented with recurrent left pleural effusion. He had history of dry cough, exertional breathlessness for the last two years. He denied any occupational exposure or second hand smoke exposure. His physical examination demonstrated decreased breath sounds on the left side of chest. Cardiac evaluation was unremarkable. Diagnostic pleural aspiration revealed straw coloured fluid, exudative, and nonmalignant in nature. CT-imaging of the chest demonstrated left pleural effusion, nodular parenchymal infiltrates in bilateral lungs, plate like atelectasis in the left lower lobe. Bronchoscopy showed diffuse airway pigmentation, right middle lobe opening, and left lower lobe bronchus pigmented and stenosed. Semi-rigid pleuro-videoscopy revealed diffuse black coloured deposits over visceral pleura and focally scattered deposits over parietal pleura. Spirometry showed mild airway obstruction with moderate impairment in diffusion capacity.

## 1. Introduction

Anthracosis is characterised by black discoloration of the tracheobronchial tree caused by the deposits of carbon, silica, and other inhaled pollutants. If anthracosis causes bronchial obstruction or obliteration, it is called as bronchial anthracofibrosis (BAF) [1, 2]. This term was first coined by Chung et al. in 1998 [3]. Anthracosis has also been reported due to nonoccupational exposure like biomass smoke, indoor air pollution, and cigarette smoke [4, 5].

Pleural involvement in BAF is a rare finding and only limited data are available. We are reporting a case of pleural biopsy proven pleural anthracosis with BAF. We thoroughly searched on pubmed and other data bases, but could find only one case of pleural anthracosis reported by Amiseno et al. from Malaysia [6].

## 2. Case Report

**Figure 1:**
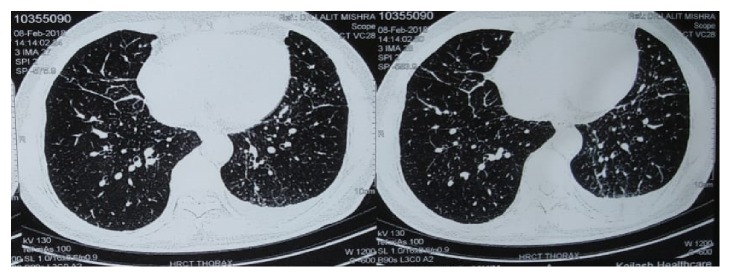
Contrast enhanced computed tomography of chest.

This is a case of a 58-year-old nonsmoker male from Ladakh in Jammu and Kashmir, India who was referred to our hospital for evaluation of recurrent undiagnosed exudative pleural effusion. He presented with breathlessness and dry cough for 1–2 years and left sided chest pain for 6 months. There was no history of fever, anorexia, weight loss, and any other constitutional symptoms. He was taking anti-tubercular treatment for pleural effusion for last six months. On general examination, vital and other parameters were normal. On respiratory examination, stony dull note was present on percussion, and breath sounds were decreased in left infra axillary and infra scapular areas. Cardiac examination was within normal limits. Routine haematological and biochemical investigations were normal. Chest radiographs demonstrated reticulo-nodular shadows and left pleural effusion. A contrast computed tomography (CT) scan (Figure 1) demonstrated bilateral multiple sub-centimetre nodules, mild interlobular septal thickening, and left sided pleural effusion with plate like atelectasis in the left lower lobe.

**Figure 2:**
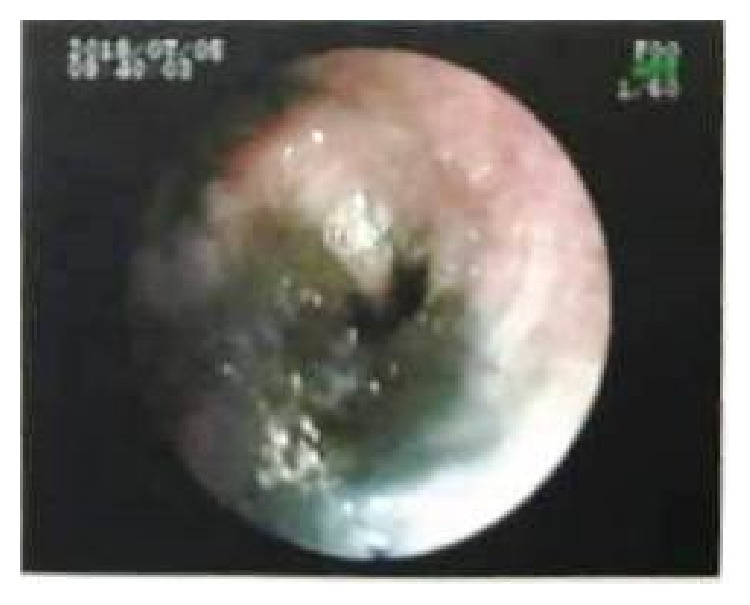
Bronchoscopy view of airway anthracosis.

**Figure 3:**
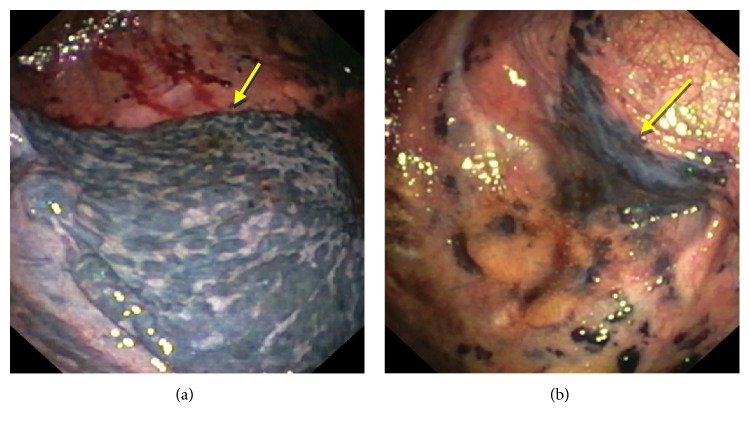
(a) Pleural anthracosis (parietal) view with thoracoscope. (b) Pleural anthracosis (visceral) view with thoracoscope.

**Figure 4:**
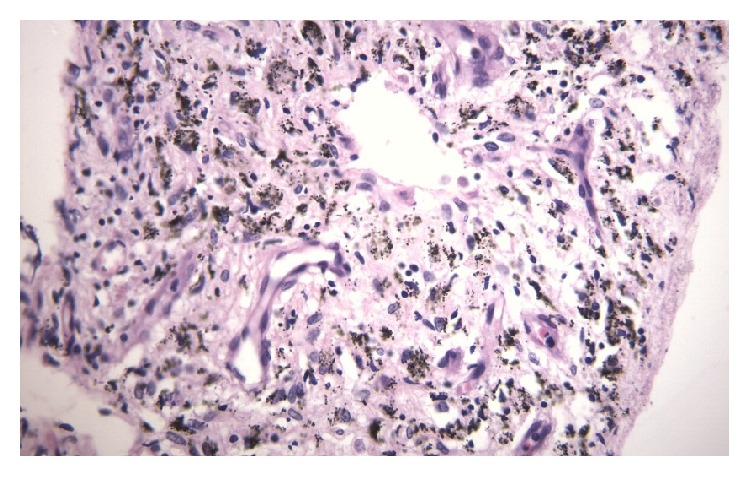
Histopathological features of pleural anthracosis.

Pleural fluid examination showed Protein-3.21 g/dl, Glucose-106 mg/dl, TLC-600 cells/cmm, Lymphocytes-99%, and Adenosine deaminase (ADA) was 5.6 IU/L. Microscopic examination revealed mainly lymphocytes against a proteinaceous background; no atypical cells were seen. In view of abnormal CECT Chest findings, flexible bronchoscopy was done, which revealed diffuse anthracotic pigmentation of mucosa in bilateral bronchial airways. Left lower lobe bronchus was narrowed due to anthracotic pigmentation (Figure 2). AFB was negative in bronchial washings and mucosal biopsy from anthracotic deposits in lungs. In view of undiagnosed exudative left sided pleural effusion, semi-rigid pleurovideoscopy was done, which revealed diffuse black coloured deposits over visceral pleura and focally scattered deposits over parietal pleura (Figures 3(a) and 3(b)). Pleural biopsy from pigmented lesions of parietal pleura was obtained. Microscopic findings of biopsy revealed nodular aggregates of macrophages with anthracotic pigment; no granulomas were seen. Histopathological examination was consistent with Pleural Anthracosis (Figure 4).

Post procedure, patient was managed conservatively with inter costal drainage tube, and discharged after complete expansion of lungs. After few days, patient again presented with left pleural effusion. In view of recurrent pleural effusion, pleurodesis was done with talc poudrage (size 5 *µ*m) through pleurovideoscope. After pleurodesis, patient was managed conservatively with inter costal drainage tube, which was removed after 3–4 days. Chest X-ray done at 1 month of follow-up, showed no pleural fluid refilling.

## 3. Discussion

Airway anthracosis is a well known bronchoscopic diagnosis which is characterized by airway mucosal hyperpigmentation which can progress to bronchial anthracofibrosis (BAF), resulting in distortion of the normal airway anatomy. Prolonged environmental exposure to smoke from burning of biomass fuel for cooking and house heating such as wood, charcoal, and animal dung cake, and certain dusts such as coal dust leads to anthracosis and BAF [7]. Repeated exposure to these compounds results in chronic inflammatory changes resulting in submucosal fibrosis and focal airway narrowing. BAF usually presents with symptoms of cough and dyspnoea [7, 8].

In India, BAF is predominantly found in elderly women in rural areas using biomass fuel for cooking purposes in poorly ventilated rooms. It has been found to be associated with respiratory diseases like tuberculosis, chronic obstructive pulmonary diseases, and pneumonia [9, 10].

Pleural anthracosis is a rare finding and only limited data are available. Earlier few case reports documented the presence of anthracotic pigment in pleural fluid, which was associated with cocaine abuse and HIV co-infection [11]. Recently, a case report of pleural biopsy proven pleural anthracosis without occupational or nonoccupational exposure to environmental smoke/dust has been reported [6]. In another study from China, 30 cases of coal worker's pneumoconiosis have been reported, who presented with pleural effusion, but none of them were diagnosed with pleural anthracosis in thoracoscopic pleural biopsy [12].

Our patient had nonoccupational environmental exposure to biomass fuel used for house heating. Ladakh in Jammu and Kashmir, India is situated at >11000 feet above the sea level. High altitude and geographic location of Ladakh leads to severe and long duration of winter with nearly sub-zero temperature every year [4]. BAF may develop due to frequent exposure to soot from the burning of wood and dung cakes for cooking and for keeping the rooms warm in winter. Due to cold climatic conditions, people usually remain indoors and keep houses at minimum ventilation.

## 4. Conclusion

Diagnosis of pleural anthracosis requires careful history including exposure to occupational/nonoccupational environmental risk factors. Many of these patients are misdiagnosed as tubercular pleural effusion and started on anti TB treatment and these patients suffer unnecessary adverse effects of anti tubercular treatment. So, exclusion of alternative diagnosis should always be done in cases of exudative pleural effusion and having history of exposure to risk factors such as burning of biomass fuel, e.g., wood, charcoal, and animal dung cake etc., for cooking and house heating and certain dusts such as coal dust. Pleural biopsy should be done to confirm the diagnosis of pleural anthracosis.
